# The mystery of persistent pulmonary hypertension: an idiopathic infantile arterial calcification

**DOI:** 10.1186/1471-2431-13-107

**Published:** 2013-07-16

**Authors:** Huma Shaireen, Alexandra Howlett, Harish Amin, Kamran Yusuf, Majeeda Kamaluddeen, Abhay Lodha

**Affiliations:** 1 Section of Neonatology, Department of Pediatrics, University of Calgary, Calgary, Alberta, Canada; 2 Foothills Medical Centre, Calgary, Alberta, Canada; 3 Alberta Health Services, Calgary, Alberta, Canada; 4 Alberta Children’s Hospital, Calgary, Alberta Canada

**Keywords:** Neonate, Infantile arterial calcification, Pulmonary hypertension, Biphosphonate

## Abstract

**Background:**

Idiopathic infantile arterial calcification (IIAC) is a rare autosomal recessive disorder, characterized by wide spread calcifications in arterial walls, leading to vaso-occlusive ischaemia of multiple organs. Mortality is high, and there is no definitive treatment.

**Case presentation:**

A male neonate, 36^+5^ weeks gestation, 2.81 kg, was admitted to NICU for respiratory distress. At one hour of age, he was noted to be pale, hypoperfused, with weak pulses, a hyperdynamic precordium and a grade IV/VI pansystolic murmur. The rest of his examination was normal. A chest X-ray showed massive cardiomegaly and pulmonary oedema. An echocardiogram (ECHO) indicated moderate persistent pulmonary hypertension (PPHN) of unclear etiology. A diagnosis of Idiopathic infantile arterial calcification was made and a trial of Editronate therapy was given without success.

**Conclusion:**

IIAC is a rare disorder, it should be considered whenever a neonate presents with unexplainable cardiac failure, PPHN, echogenic vessels on X-ray/ultrasound and, or concentric hypertrophic ventricles on ECHO. Serial antenatal ultrasound findings of echogenic cardiac foci should raise the suspicion of IIAC. Further studies to determine the long term effects of Editronate on vascular calcifications, disease outcome, and other treatment options are needed.

## Background

Idiopathic infantile arterial calcification (IIAC) is a rare autosomal recessive disorder associated with widespread calcification and degeneration of the elastic lamina of arteries [1]. Clinical manifestations are related to vaso-occlusive disease of multiple organs [2].The association of IIAC with persistent pulmonary hypertension (PPHN) is rare [3,4]. We describe a newborn with IIAC who presented with PPHN and cardiac failure.

## Case presentation

A male neonate, 36^+5^ weeks gestation, 2.81 kg, was admitted at birth to neonatal intensive care unit (NICU) for respiratory distress. The family history was notable for parental consanguinity. He was delivered to a G2 P1 mother after an uncomplicated pregnancy by emergency caesarean section for fetal heart rate abnormalities. Apgar scores were 9^1^ and 9^5^, with no resuscitation required. At one hour of age, he was noted to be pale, hypoperfused, with weak pulses, a hyperdynamic precordium and a grade IV/VI pansystolic murmur. The rest of his examination was normal. A chest x-ray showed massive cardiomegaly and pulmonary oedema. An echocardiogram (ECHO) indicated moderate PPHN. He required intubation, and treatment with inhaled nitric oxide (iNO), sildenafil, furosemide and milrinone.

Complete blood counts, electrolytes, blood cultures, enterovirus panel, metabolic investigations and cardiac enzymes were normal. Molecular genetic testing for Myoclonic epilepsy with ragged red fibers (MERRF), Mitochondrial encephalomyopathy, lactic acidosis, and stroke-like episodes (MELAS) and Neuropathy, Ataxia, and Retinitis Pigmentosa (NARP) syndrome was negative. Repeated ECHO examinations showed normal systemic and pulmonary venous drainage, thickened semilunar and tricuspid valve leaflets, and concentric left ventricular hypertrophy. An ECG was negative for ischaemic changes. Computed chest angiography showed incidental mild narrowing of the left pulmonary vein, which was not noticed in any of the ECHO examinations.

**Figure 1:**
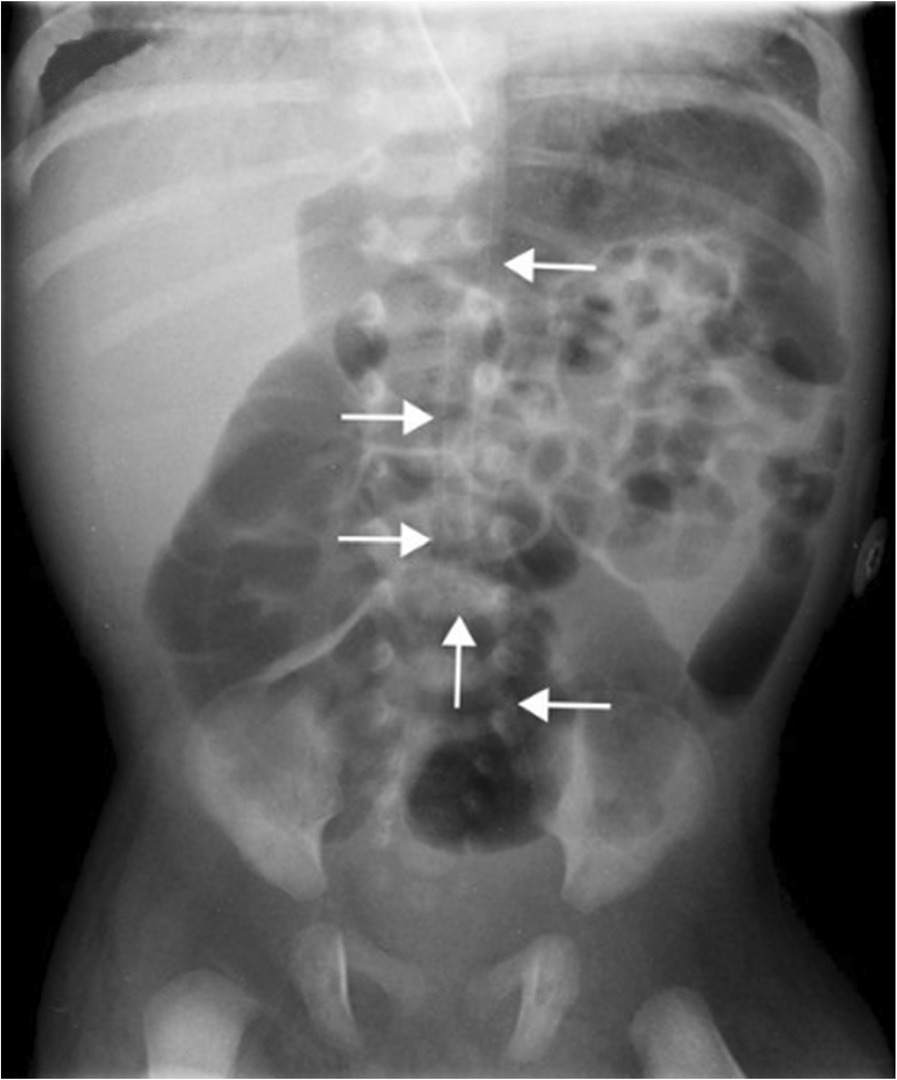
**Abdominal X**-**ray showing calcified descending aorta.**

**Figure 2:**
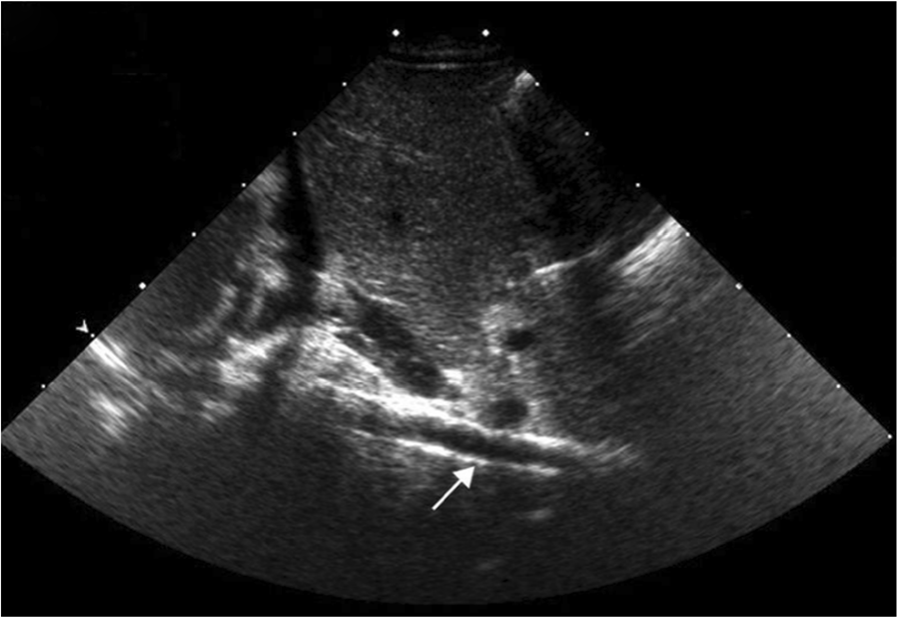
Ultrasound of abdomen showing calcified aorta.

A detailed review of an abdominal X-ray (Figure 1), and computed tomography (CT) scan found calcified vessels, suggested the clinical diagnosis of IIAC on day 20 of life. An ECHO on day 20 of life also identified an echogenic aorta, echogenic coronary arteries with normal pulmonary veins. Echodensities were also noticed in the main and branch pulmonary arteries. Calcifications of the abdominal aorta (Figure 2), renal artery and intracranial thalamic vessels were also evident on ultrasound. No mutation of ectonucleotide pyrophosphatase/phosphodiesterase 1 (ENPP1) gene was identified. Blood was stored for DNA banking to identify mutations in other genetic sequences.

The baby was extubated on day 20, iNO and milirinone were discontinued gradually. The Sildenafil was tapered to 1 mg/kg/day. The infant’s subsequent course in NICU was complicated by recurrent abdominal distension, feeding intolerance, irritability, pyrexia and systemic hypertension.

Oral Editronate Sodium (10 mg/kg/day, twice a day) was started on day 40. He was discharged home at 75 days of age on Editronate 20 mg/kg/day; Sildenafil 1.5 mg/kg/day; and Captopril 1.2 mg/kg/day. He developed fever and respiratory distress one day after discharge, requiring readmission. The respiratory viral panel, and sepsis work up were normal but troponin was elevated (0.53 ug/L, normal value 0.0-0.1 ug/L). Twelve leads ECG was not available but a tracing showed S-T segment elevation in lead II. Resuscitation code was revised. Palliative care was initiated with parental consent, and no escalation of pulmonary hypertension therapy was advised. Despite ventilatory and inotropic support for hypotension, he died at three months of age. The parents declined consent for autopsy, skin/arterial biopsy and sibling genetic screening.

## Discussion

IIAC was first described in 1901 [1]. It is clinically and genetically a heterogeneous disease [2,5,6]. The most described pathogenesis in 80% of cases is a mutation and inactivation of ectonucleotide pyrophosphatase/phosphodiesterase 1 (ENPP1) gene [7-11]. ENPP1 is expressed on fibroblasts, osteoblasts and hepatocytes [7]. ENPP1 gene has nucleotide pyrophosphohydrolase activity and produces inorganic pyrophosphate (PPi) [11]. PPi prevents deposition of calcium hydroxyapatite crystal in the arteries. The mutation of ENPP1 gene results in variable degrees of calcification and intimal fibrosis of medium and large arteries of cardiac and renal vascular system [9]. There are other known (ABCC6, NT5E and SLC 20A2) and unknown gene mutations, associated with arterial calcification [5,12,13].

A literature review stated that almost 48% of patients presented at birth or in-utero, while 52% cases were identified during infancy [1]. Fetal complications include: cardiac arrhythmias, heart failure, hydrops fetalis and fetal demise, often early in pregnancy [2,14,15]. During the neonatal period, 30-40% of infants presented with respiratory or cardiac failure [1]. Seizures, renal failure, hypertension, arrhythmias and gut obstruction/infarction were seen in 1% to 8% of neonates [1]. In infants, common manifestations include: irritability, feeding difficulties and poor growth [1]. Coronary artery calcification was the worst prognostic indicator and majority of infants expired due to myocardial infarction [1,16].

IIAC is a diagnosis of exclusion [2]. Absence of other infantile arterial calcification disorders, normal serum calcium/phosphrous panel and a positive family history suggest IIAC [1]. The gold standard for diagnosis is arterial biopsy [1,8,10,17]. Evidence of calcified arterial walls on X-rays of the chest/abdomen and long bones, CT of chest, ECHO, cranial and abdominal ultrasound are other helpful diagnostic findings [2,8].

Antenatal screening can be done by DNA analysis for ENPP1 mutation and by serial ultrasounds [15]. Detection of echogenic intracardiac foci, arterial calcification, dilated or hypertrophied cardiac chambers, hydrops fetalis are strong indicators of IIAC [14]. Families can be screened for calcified arteries by performing ultrasounds, X-rays and echocardiography [15].

In the literature only few cases of IIAC with PPHN have been reported [4,10]. One patient received Editronate 15 mg/kg/day, but had remained hypertensive, ventilator dependant and died at 35 days of life [10]. Extensive calcification of major vessels was confirmed on autopsy. Our patient also had PPHN with other systemic signs attributable to IIAC.

Reports in the literature suggest resolution of arterial calcification with Editronate (Biphosphonate sodium) treatment [8,18]. The longest survival has been reported in a 25-year-old man who was treated with Editronate (Biphosphonate sodium) [1]. However, all patients who received Editronate had mild or no evidence of coronary involvement. Editronate is an analogue of PPi [8]. It alters the calcium balance by interfering with hydroxyapatite crystal formation and inhibits the calcium deposition in the existing calcified lesions. The recommended dose and duration of this treatment varies in the literature [8,18]. Our patient was maximized to 20 mg/kg/day, twice a day. Serial calcium, phosphate, alkaline phosphatase and wrist X-rays were done to monitor the side effects of the drug [18,19]. Vitamin D was also given to prevent Editronate induced vitamin D deficiency. No resolution of calcification was seen in the serial radio-imaging studies.

Our patient also had coronary artery calcification, a common cause of sudden ischemic heart failure [1]. We assumed that myocardial ischemia was the probable cause of death because of high troponin levels. Arterial biopsy and autopsy were not performed as per parent’s wishes.

## Conclusions

Though IIAC is a rare disorder, it should be considered whenever a neonate presents with unexplainable cardiac failure, PPHN, echogenic vessels on X-ray/ ultrasound and, or concentric hypertrophic ventricles on ECHO. Serial antenatal ultrasound findings of echogenic cardiac foci should raise the suspicion of IIAC. Every effort should be made to establish the diagnosis by arterial and skin fibroblast biopsy. Further studies to determine the long term effects of Editronate on vascular calcifications, disease outcome, and other treatment options are needed.

### Consent

Written informed consent was obtained from the patient for publication of this case report and any accompanying images. A copy of the written consent is available for review by the Editor of this journal.

## Abbreviations

ENPP1: ectonucleotide pyrophosphatase/phosphodiesterase 1; ABCC6: ATP-binding cassette (ABC) transporters; NT5E: 5'-nucleotidase, ecto (CD73) and; SLC 20A2: SLsolute carrier family 20 (phosphate transporter), member 2.

## Competing interest

There are no competing interests in the report.

## Authors’ contributions

HS Has written the first and revised draft of the manuscript, provided patient care in the NICU and approved the final manuscript as submitted. AH Reviewed and edited the manuscript, and approved the final revised manuscript as submitted. HA Critically reviewed the manuscript and approved the final revised manuscript as submitted. KY Provided patient care, helped in the literature search, reviewed the manuscript and approved the final revised manuscript as submitted. MK Reviewed the manuscript and approved the final revised manuscript as submitted. AL Provided patient care, obtained consent for publication and formatted bibliography, Reviewed and revised the first draft and approved the final revised manuscript as submitted.

## Authors’ information

HS. MD, Clinical Fellow (4^th^ year), University of Calgary, Calgary, Canada

AH. MD, FRCPC, FAAP, Clinical Associate Professor, Department of Pediatrics, University of Calgary, Chief of Neonatology, Alberta Children’s Hospital, Calgary

HA. MD, FRCPC, FAAP, Associate Professor, Department of Pediatrics, University of Calgary, Chief of Neonatology, South East Hospital, Calgary.

KY. MBBS, FAAP, Assistant Professor, Department of Pediatrics, University of Calgary

MK. MBBS, MD, MRCPI, FAAP, Director, Neonatal- Perinatal Medicine Fellowship Program, University of Calgary.

AL. MBBS, MD, DM, MSC, Chairman CME, Epidemiologist, CME, Assistant Professor, Department of Pediatrics and Community Health Sciences, University of Calgary.

## Pre-publication history

The pre-publication history for this paper can be accessed here:

http://www.biomedcentral.com/1471-2431/13/107/prepub
